# Do patient engagement interventions work for all patients? A systematic review and realist synthesis of interventions to enhance patient safety

**DOI:** 10.1111/hex.13343

**Published:** 2021-08-25

**Authors:** Bronwyn Newman, Kathryn Joseph, Ashfaq Chauhan, Holly Seale, Jiadai Li, Elizabeth Manias, Merrilyn Walton, Stephen Mears, Benjamin Jones, Reema Harrison

**Affiliations:** ^1^ Centre for Health Systems and Safety Research (CHSSR), Australian Institute of Health Innovation Macquarie University Sydney New South Wales Australia; ^2^ School of Nursing and Midwifery, Centre for Quality and Patient Safety Research, Institute for Health Transformation Deakin University Geelong Australia; ^3^ Faculty of Medicine and Health, School of Population Health University of New South Wales Sydney New South Wales Australia; ^4^ School of Public Health The University of Sydney Sydney New South Wales Australia; ^5^ Hunter New England Health Libraries, John Hunter Hospital, HRMC Newcastle New South Wales Australia

**Keywords:** patient engagement, patient participation, patient safety, point of care, systematic review

## Abstract

**Background:**

Patients are increasingly being asked for feedback about their healthcare and treatment, including safety, despite little evidence to support this trend. This review identifies the strategies used to engage patients in safety during direct care, explores who is engaged and determines the mechanisms that impact effectiveness.

**Methods:**

A systematic review was performed of seven databases (CINAHL, Cochrane, Cochrane‐Central, Embase, ISI Web of Science, Medline, PsycINFO) that included research published between 2010 and 2020 focused on patient engagement interventions to increase safety during direct care and reported using PRISMA. All research designs were eligible; two reviewers applied criteria independently to determine eligibility and quality. A narrative review and realist synthesis were conducted.

**Results:**

Twenty‐six papers reporting on twenty‐seven patient engagement strategies were included and classified as consultation (9), involvement (7) and partnership (11). The definitions of ‘patient engagement’ varied, and we found limited details about participant characteristics or interactions between people utilizing strategies. Collaborative strategy development, a user‐friendly design, proactive messaging and agency sponsorship were identified as mechanisms to improve engagement about safety at the point of direct care.

**Conclusions:**

Agency sponsorship of collaboration between staff and patients is essential in the development and implementation of strategies to keep patients safe during direct care. Insufficient details about participant characteristics and patient–provider interactions limit recommendations for practice change. More needs to be learned about how patients are engaged in discussions about safety, particularly minority groups unable to engage with standard information.

**Patient or Public Contribution:**

Review progress was reported to the CanEngage team, including the consumer steering group, to inform project priorities (PROSPERO CRD42020196453).

## 1 BACKGROUND

Patients are often the only constant element in their healthcare journey and provide important contextual information for designing safe healthcare services.^1^ The fact that patients can retrospectively identify unsafe events that occurred during their care is well established.^2^, ^3^, ^4^, ^5^ They also play an active role in their own safety by raising concerns or flagging inconsistencies and inaccuracies during healthcare interactions.^4^ Over the past 20 years, interventions that encourage patients to discuss or raise concerns about inaccuracies relevant to their care have been implemented.^6^, ^7^, ^8^ These interventions occurred simultaneously with evaluations of patient involvement in system‐ and service‐level patient safety programmes, such as patient‐led incident reporting systems.^9^ The use of patient‐centred tools and strategies to enhance safety has increased despite limited evidence about their effectiveness.

Limited research about the effectiveness of patient engagement, and the depth of engagement needed to promote safe care, is reflective of wider inconsistencies. There are various definitions of patient engagement, involvement and participation in the literature.^10^ Carman et al.^10^ defined patient engagement as ‘patients, families, their representatives, and health professionals working in active partnership at various levels across the health care system’ (p. 224). The framework of engagement developed by Carman et al.^10^ builds upon Arnstein's^11^ work and classifies patient engagement across a continuum, ranging from patient consultation through to partnership. This engagement continuum spans three distinct spheres of patient engagement: direct care, organisational design and governance and policy making. In light of the variation in what constitutes patient engagement present in current research and practice, Carman et al.'s^10^ definition and framework are used throughout this review. This paper focuses on engagement strategies implemented in the ‘direct care’ sphere of engagement. Carmen et al.'s^10^ sphere of direct care aligns with the clinical point of care and refers to the period when clinicians deliver healthcare services or treatments to patients; this can be hospital or community based.^12^

With growing recognition of the value of engaging patients in healthcare design and delivery, and the susceptibility that some specific population groups have to adverse events, the need for better data about facilitating engagement is imperative. The literature identifies various system, service and clinical factors that support effective patient engagement such as education about their condition,^13^, ^14^ empowerment to engage^15^, ^16^ and the willingness and ability of clinicians and patients to communicate about safety.^17^, ^18^, ^19^, ^20^ The extent to which an organisation is committed to patient engagement is a measure in most organisational safety culture surveys, but there is little evidence of the enablers and system prerequisites to facilitate effective engagement.^21^, ^22^ Evidence of the enablers and system prerequisites for effective engagement have not been synthesized to support the implementation of such interventions.^22^ Similarly, evidence about the nature and extent to which patients are engaged in safety is fragmented and lacks information about approaches for diverse populations, such as people from culturally and linguistically diverse (CALD) backgrounds or other communication needs.^8^, ^21^, ^23^, ^24^

This systematic review aims to address the knowledge gaps identified above using a realist synthesis^25^ to explore the following questions: (1) What interventions have been used to engage patients in safety during direct care and what is the mode of intervention (e.g., video, paper chart, electronic portal) and extent of engagement (e.g., number of opportunities, with whom)? (2) What types of patients and their contexts are described in the interventions? (3) What are the mechanisms that influence the effectiveness of consumer engagement approaches in enhancing safe care and treatment?

## 2 METHODS

A systematic review and realist synthesis were undertaken and reported in accordance with the Preferred Reporting Items for Systematic Review and Meta‐Analyses (PRISMA) statement.^26^

### 2.1 Prospero registration number: CRD42020196453

*Inclusion criteria*: Studies published between January 2010 and December 2020 in English were included. All research designs were eligible, including qualitative, quantitative, multi‐ and mixed‐method studies. All studies included participants who were healthcare consumers, patients, family members or other caregivers. Safety outcomes in the clinical encounters described encompassed increased notifications of or the prevention of safety breaches, errors, accidents, incidents, complications and infections. Selected interventions had to use patient engagement designed to minimize harm. Studies that did or did not include a comparator intervention were eligible.

*Exclusion criteria*: All studies outside the date range or published in a language other than English were excluded. Systematic or other literature reviews were not included, but their reference lists were searched. Studies that focused on methods beyond direct care, for example, to enhance governance or inform improvements to organisational safety for example, adverse event reporting systems or service governance, service planning, self‐management or improving health, such as self‐management for people with chronic conditions, were beyond the scope of the present review. Studies about patient involvement in training medical or nursing staff, patient attitudes towards safety or willingness to participate and studies about participatory research or codesign methods unrelated to safety were not included.

### 2.2 Study identification

The key concepts of patient engagement and unsafe healthcare were used to generate keywords, synonyms and phrases to inform a comprehensive search strategy (see File S1). The search strategy was applied to seven databases: CINAHL, Cochrane, Cochrane‐Central, Embase, ISI Web of Science, Medline, PsycINFO January 2010 and December 2020. In addition to searching the reference lists of the included studies, hand searches of the following relevant journals were conducted to locate further potentially recently published eligible studies: *The Journal of Patient Safety*, *The British Medical Journal of Quality and Safety*, *The International Journal for Equity in Healthcare*, *BMC Health Services Research* and *The International Journal for Quality in Healthcare*.

### 2.3 Study selection and data extraction

Search results were exported to Endnote (X10) and duplicates were removed. Articles were then extracted to Covidence systematic review management software (Veritas 150 Health Innovation). Two reviewers (J. L. and B. J.) completed the initial title and abstract review, followed by an independent screening by a third reviewer (B. N). The inclusion criteria were then independently applied to full‐text articles by two reviewers (B. N. and R. H.), with disagreements or uncertainty resolved through discussion. The following data were extracted: author, year, country, aims/objectives, setting, number of participants, participant characteristics, inclusion of diverse populations, method of data collection and samples, intervention/method of patient engagement, main findings and what worked (enablers, barriers).

### 2.4 Assessment of study quality

Due to the heterogeneity of the study types, the Quality Assessment Tool for Diverse Studies, a validated quality appraisal tool,^27^ was used. Two reviewers (B. N. and K. J.) independently applied the 13 criteria to the included studies. The *κ* test was used to determine inter‐rater reliability, and substantial reliability was confirmed (*κ* = .726).^28^

### 2.5 Data synthesis

Findings were synthesized using a narrative approach and the realist framework to explore which interventions worked, in what conditions and with whom.^25^ Realist evaluation was selected because it examines the conditions that facilitate success rather than just information about whether the outcome or intervention ‘worked’.^29^

Key findings relevant to the review questions were extracted, including barriers and enablers to implementation. Carman et al.'s^10^ engagement framework was used to determine the extent of engagement in the interventions, and engagement strategies are described in relation to the three levels of engagement.

## 3 RESULTS

**Figure 1:**
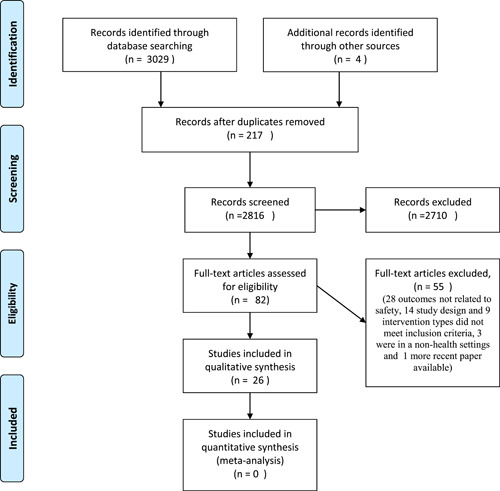
Preferred Reporting Items for Systematic Review and Meta‐Analyses 2009 flow diagram

The systematic search produced 3029 papers, with 2706 studies excluded and 217 duplicates removed. A total of 82 full texts were reviewed and 55 were excluded (28 included outcomes not related to safety, 14 study designs and 9 intervention types did not fulfil the inclusion criteria, 3 were in nonhealth settings and 1 more recent paper available), leading to 26 included publications describing 27 strategies. Figure 1 shows the search and selection process.

*Characteristics of the included studies*: Studies originated from the United States (13), the Netherlands (4), the United Kingdom (3), Canada (2), Vietnam (1), Australia (1), Korea (1) and Norway (1). Seventeen of the twenty‐six studies were focused on inpatient safety, three on specific clinics or treatments and six were focused on treatment between face‐to‐face visits. The studies were conducted in a range of clinical areas including inpatient adult general medical services,^30^, ^31^, ^32^, ^33^, ^34^, ^35^, ^36^, ^37^, ^38^, ^39^ inpatient surgical departments,^40^, ^41^, ^42^ adult oncology,^43^ outpatient radiology clinics,^44^, ^45^ adult intensive care,^46^ residential aged care facility,^39^ inpatient paediatric services,^34^, ^45^, ^46^, ^47^, ^48^, ^49^, ^50^, ^51^ with two whole of hospital studies^52^, ^53^ and homecare visits, appointments, discharge and pharmacy community.^32^, ^52^, ^54^

*Study quality*: A score of 0–3 was assigned in the 13 categories used to assess quality (see File S2). Of the 26 included papers, most achieved high scores (2 or 3) in the categories of statement of aims (23), appropriate study design to address research aims (22) and format of data collection tool to address research aims (23). Fourteen studies contained limited details in the participation data provided (Criteria 9) and eight described involvement of ‘consumers or stakeholders’ (Criteria 12) in the process of study design and conduct.

### 3.1 Review findings

#### 3.1.1 What interventions have been used to engage patients in safety activities during direct care, and what is the mode of intervention and extent of engagement?

Twenty‐seven patient engagement strategies were reported in twenty‐six publications (Table 1). The engagement activities are described in relation to approaches that were focused on consultation, involvement or partnership relative to the Carman Framework.^10^ Evidence from each study of the effectiveness of a strategy in improving safety is presented in Table 1.

**Table 1:** Summary of the study findings

Author	Year	Country/setting	Aims/objectives	Method/sample	Intervention type	Main findings	Enablers	Barriers
Baker et al.^52^	2016	Canada/hospital and community	To evaluate three efforts to engage patients in quality improvement efforts	Case study	PARTNERSHIP	By implementing patient‐informed practices (TCAB), they achieved a 25% decrease in Clostridium difficile and vancomycin‐resistant Enterococci infections. Identified that	*Client‐centred systems at many levels of care and governance. *Opportunities for patients and staff to work together towards tangible goals. *Cultural shift in valuing patients, families and *caregivers as partners. * peer mentors, *multiple participating advisors, *external facilitators for events, *leadership support and role modelling	None identified
					Transforming care at the bedside (TCAB) project to increase quality of bedside interaction			
				Case study	PARTNERSHIP Collaboratively developed a hip surgery post‐op complication kit that encouraged engagement	The FReSH START Toolkit: led to better satisfaction and proactive approach to prevent complications		
Bell et al.^53^	2017	USA/hospital	To evaluate a new patient reporting tool named OpenNotes focused on safety concerns	9‐Question survey and designed a FAQs document for patients (*n *= 2736)	INVOLVEMENT	Giving patients/care partners the opportunity to review online notes can increase safety for the individual and improve practices. Concerns raised in 59 reports (23%), most commonly possible mistakes (21%)	*Access to information provided greater transparency, and did not impact the relationships between providers and service users	Health literacy, cultural differences, demographic factors, cognitive issues and provider‐related factors
					OpenNotes patient feedback reporting tool			
Bergal et al.^40^	2010	USA/hospital	Strategy to improve the safety and quality of care, promote patient education and provide a tool for a cooperative treatment approach	Patients to mark the site of surgery to see if this helped avoid wrong‐site surgery (*n *= 135)	CONSULTATION	68.2% Compliance—concluded that patient involvement in surgical site marking is unreliable and may not help in decreasing the chances of wrong‐site surgery. Of the 200 patients who were enroled, 135 made the mark	*Younger participants more ‘compliant’ and engaged in their care. *Asking the patient to mark the site when not sedated/medicated, appropriate timing between education and surgery (not too long before)	*Age and cultural beliefs impacted compliance *difficult to measure impact of study as wrong‐site surgery is uncommon
					Asking patients to mark on their body where surgery was to occur			
Buning et al.^30^	2016	The Netherlands/hospital	To examine the availability and accountability of a web tool for medication coordination (MMa) during medical transitions and across various appointments/points of care	Patients completed the MMa list on existing portal and compared with a list of meds completed by pharmacists, questionnaire (*n* = 17)	PARTNERSHIP	Discrepancies between the lists were differences in dose and frequency (*n* = 27) and differences in medications registered (*n* = 15). *Patients had a more accurate list that the list compiled by the pharmacy practitioner. *Mobile application was feasible and well accepted by the patients	*Skilled tech users more likely to participate—not clear whether they would be more likely to use app. Or take part in research *patients were positive about it controlling data across sites	Patients would have preferred password feature for security
					Web‐based tool for patients to be part of medication reconciliation at points of transition—for use in various care settings. Patient owns and is responsible for the information rather than a different system at each service/doctor's visit			
Campbell et al.^51^	2020	Vietnam/hospital	To measure the effectiveness of visual empowerment tools to improve hand hygiene in Southeast Asia	Observation before and after intervention (*n *= 73)	PARTNERSHIP	Giving families visual reminder tools and brief education is associated with increased HCW HH. Baseline HH adherence was 46%, and increased to 73% during the implementation period (*p* < .001)	*Low‐cost tool *empowerment and education of patients rather than education of HCW. *Leaving a visual reminder at the bedside enabled patients to show a reminder rather than confront HCWs	Fear of HCW reaction
					Visual tool to empower patients to remind healthcare workers to wash their hands and script for use by the research assistant to explain how to use the visual tool			
Cox et al.^47^	2017	USA/hospital	To examine the impact of the family‐centred rounds (FCRs) checklist and associated provider training, on performance of FCR elements, family engagement and patient safety	Parent survey, admission data for length of stay and video recording of FCRs (*n *= 273)	PARTNERSHIP	Using the checklist enhanced engagement and impacted safety of care. Safety findings—measured climate but commented on increased awareness of hand hygiene and open communication during handover re. meds	*The checklist incorporates recommendations for FCRS with non‐English speakers. *Reading back ‘orders’ may be useful to foster safe medication use	*Stress *new diagnosis, *English proficiency impacted research participation. But these are not necessarily barriers for use of FCR
					Family engagement in rounds—checklist for families to measure 8 elements. Data analysis = qualitative review of video footage, survey of families was conducted re. safety			
de Jong et al.^38^	2016	The Netherlands/community	To investigate whether quality of the electronic medication record improves when patients play a vigilant role	Digital questionnaires and patients visit the pharmacist twice for verification consultations (*n *= 152)	INVOLVEMENT	Forty‐nne percent of participants never logged into their eMAR. Corrections were necessary for approximately 20% of eMARS because the patient started/stopped a medication, changed the timing of medication and changed dosage	*User‐friendly *daily users of the internet were more likely to email	Data collection process was onerous
					Electronic medication administration record (eMAR) patient communication tool. Module on the pharmacist's website with a personal patient log‐in. Patients can view their prescribed medications (inc. method, dose, freq.) and communicate with the pharmacist regarding errors. Patients were invited to check after every change to prescription			
Duckworth et al.^31^	2019	USA/hospital	To assess the effectiveness for engaging patients and family in the three‐step fall prevention process using varied modalities: electronic toolkit, laminated toolkit and bedside display	Randomly selected patients asked what their plan is and nurse checks if there is a plan visible at bedside (*n *= 1209)	CONSULTATION	Providing three Fall TIPS modalities is effective and flexible approach. Over 80% adherence with protocol. It is possible to have evidence‐based falls programme within current workflow and to engage patients in any of the modalities	*Support from leadership *good communication channels *flexibility in the way sites can automate/implement	*Lack of systemic support, *lack of funding, *limited staff engagement
					Examining a previously developed intervention adding modalities—a falls prevention kit that is electronic, laminated and a poster			
Dykes et al.^46^	2017	USA/hospital	To examine the efficacy of a patient‐centred care and engagement programme implemented in ICUs. To shift the clinical paradigm from providers alone determining ‘What is the matter?’ to discovering ‘What matters to you?’	Whole of hospital, reported experience/satisfaction and adverse events‐ survey in person before transfer from ICU, phone survey by research staff 45 days postdischarge (random sample) (*n *= 2105)	PARTNERSHIP	Reduction in adverse events and improved patient and care partner satisfaction. Adverse events decreased 29% 59.0 per 1000 patient days (95% CI: 51.8–67.2) to 41.9 per 1000 patient days (95% CI: 36.3–48.3; *p* < .001), during the intervention period of 11 months compared to the baseline period (preceding 12 months)	*Patient‐centred care, *staff training, *staff skill/attitude, *partnership with care partners (as the patient is often too ill)	*Portal was most used by white, young privately insured patients, *activating portals is complex
					Structured patient‐centred care and engagement training programme and web‐based technology including ICU safety checklist, shared care plan and messaging platform. Patients can access the online portal to view health information, participate in the care plan and communicate with providers			
Dykes et al.^55^	2020	USA/hospital	To assess whether a fall prevention toolkit that engages patients and families in the fall prevention process throughout hospitalisation is associated with reduced falls and injurious falls	Falls data and injury from falls data per 1000 patient days (*n *= 37,231)	CONSULTATION	Engaging patients in fall‐prevention was associated with fewer falls among younger patients and substantially fewer fall‐related injuries among older patients. There was a 34% reduction in falls after implementation	*Team approach, *engagement of hospital leadership staff, *continuous engagement	*Timing, *limited engagement of leadership staff
					Nurses reviewed tailored falls TIPS with the patient at admission and each shift			
Ekstedt et al.^43^	2014	Norway/hospital and community	To explore if and how an e‐message service, a single component of practice‐integrated SMSS, affect the continuity of care and safety of patients with breast cancer	Data collected through an online tool WebChoice (*n *= 200)	PARTNERSHIP	Safety related to messages about information double checking and coordination of care. Ninety‐one dialogues consisting of 284 messages were analysed. Patients were able to clarify, report concerns more easily and ensure accurate transfer of information between services	*Informed patient seen as a buffer against medical mishaps and delays, *giving patients opportunities to check on advice that they had been given, ask specific questions	*Researchers identified that new tools may increase the complexity of interactions
					Online self‐management support system (SMSS) e‐messaging service. Patients can direct questions to nurses, physicians and social workers			
Garfield et al.^32^	2020	UK/primary and secondary healthcare	To identify mechanisms by which patient‐held medication lists could be used to support safety, key supporting features and barriers and facilitators to their use	Two focus groups with patients and carers, 16 interviews with healthcare professionals (*n* = 16), interviews with people carrying medication lists (*n *= 60), quantitative features analysis of tools and usability testing of four tools	PARTNERSHIP	Patients and healthcare professionals perceive patient‐held medication lists to have a wide variety of benefits, need a variety of options, useful especially in transitions and emergencies	*Support from healthcare professionals, family/friends *patients recognizing need—trigger event for example, emergency or extra complexity of med schedule *flexibility, *clear purpose of the tool	*Lack of patient awareness that health systems were not linked—assumed that health staff already knew about meds. *Small number of patients were concerned about confidentiality, *bulky paperwork
					Explored the use of various existing patient‐held medication tools (103 tools). Electronic and paper‐based tools were included			
Gerard et al.^33^	2017	USA/hospital	To learn more about patient experiences with reading and providing feedback on their visit notes	Electronic portal for patients to review notes. Two‐hundred and sixty reports	INVOLVEMENT	Access to notes gave patients the opportunity to confirm what they needed to do, gain access to info faster, share info with others, engage with clinicians. Identified the benefit for correcting mistakes and inaccuracies	*Education level, *comfort using technology, *giving patient access to information about their health, *making EHR more open and interactive	*Limited confidence with technology
					Patients can use the open notes portal to engage with electronic notes. This study examined what they value about that tool as part of a bigger quality improvement project			
Grossman et al.^34^	2018	USA/hospitals and medical centres	To provide recommendations on how to most effectively implement advanced features of acute care patient portals	Case studies‐ descriptive exploration of 6 sites that use patient portals (*n *= 1065)	INVOLVEMENT	Need to adjust current portals to meet stakeholder needs—patients and providers	*Having an ‘invite’ feature could eliminate privacy issues, *consistency in how info is reported, *photographs and bios of care team, *having communication categories for feedback and communication in addition to generic messaging features	*Practitioners fear interruption to workflow *privacy issues re. caregivers viewing info
					Various interventions across 6 sites			
Heyworth et al.^35^	2014	USA/hospital at discharge	To test the use of an online medication reconciliation tool ‘Secure Messaging for Medication Reconciliation Tool’ (SMMRT) among patients to improve patient safety	Patients viewed medications and clarified any inaccuracies through SMMRT and 10 in‐depth interviews (*n *= 60)	INVOLVEMENT	Sent 51 SMMRT messages to patients and received 34 replies. In 40/51 patients, there was a discrepancy between the discharge summary and medications filled in at the pharmacy. This amounted to 108 clinically important discrepancies total. 68% were medication omissions an 19% were medication duplications. 23 potential Adverse drug events were identified; 43% were potentially life threatening	*Technical support, *easy to use, *rapid access to health info	*No computer access or knowledge of the internet, *patients may have meds prescribed elsewhere that are not part of the study
					An online portal; patients view their medications in a secure email message and reply using SMMRT's interactive form to verify medication regimes and clarify inaccuracies			
Khan et al.^48^	2018	USA/hospital	To determine whether medical errors, family experience and communication processes improved after implementation of an intervention to standardize the structure of healthcare provide–family communication on FCRs	AEs and medical errors were measured using an established tool to determine the number of events per 1000 patient days (*n *= 3106)	PARTNERSHIP	Although overall errors were unchanged, harmful medical errors decreased and communication processes improved	*Staff training, *collaborative development of tool, *structured communication framework, *iterative development with family involvement, *using plain language, *structural change to accommodate rounds	*Before intervention—family passive at sites that did have bedside rounds, some had rounds without patients present, jargon was used, social issues or limited English often precluded involvement in rounds
					The intervention consisted of a communication framework for rounds, family engagement and bidirectional communication principles			
Kim et al.^44^	2017	Korea/outpatient clinic	Verify the effectiveness of patient involvement in identifying both patients and the location(s) before X‐ray examinations at orthopaedic clinics	Over 1 year, 2013−2014, using comparison of errors before and after intervention. Errors were categorized and analysed. *n* = 13,617 (Group 1) and *n* = 12,588 (Group 2)	CONSULTATION	There was a significant reduction in errors. The rate of X‐ray prescription errors declined from 0.58% (Group I, 79/13,617) to 0.08% (Group II, 10/12,588)	*Simple change to practice	*Staff change was problematic—need consistent data collector
					Staff asking additional questions of patients to confirm the X‐ray site			
Lachman et al.^49^	2015	UK/hospital	Testing and introduction of a self‐reporting, real‐time bedside tool	The reporting tool was developed in three stages: (1) tested patient readiness with a questionnaire, (2) designed a patient‐centric process for managing risk with a real‐time daily reporting tool and (3) staff checked the tool each day at the end of their shift (*n = *85)	PARTNERSHIP	Thirty events were recorded, the highest proportion almost equally relating to equipment problems and communication issues. During this study period, staff critical incident reporting increased to 2.31 reports per week. Only 3% of incidents reported by patients were reported in the staff reporting process	*Pictorial reporting tool did not require high levels of literacy or English proficiency—children could also use it, *patient engagement throughout and using visual tool, *proactive approach rather than only reactive	*Questionnaire was time‐consuming and reliant on English‐language proficiency, *involving staff in the reporting process leads to fewer reports
					Daily safety reporting tool and questionnaire			
Lawton et al.^50^	2016	UK/hospital	To evaluate the efficacy of the Patient Reporting and Action for a Safe Environment intervention	PMOS 44‐point questionnaire, PIRT = safety reporting tool for patients. Both are validated tools. Average of 25 patients per ward at three‐time points	CONSULTATION	Intervention uptake and retention of wards were 100% and patient participation was high (86%), no significant effect of the intervention on any outcomes at 6 or 12 months. However, for those for which the wards were directly accountable, intervention wards showed improvement compared with control wards	*Hospital ward engagement	*Development and adherence to action plans
					The ward‐level intervention/data collection comprised two tools: (1) a questionnaire (patient measure of safety (PMOS)) and (2) a proforma for patients to report both safety concerns and positive experiences (patient incident reporting tool)			
Lutjeboer et al.^45^	2015	The Netherlands/outpatient clinic	To compare patient safety and patient satisfaction between patients who were subjected to a preprocedural visit to an IR outpatient clinic	Safety checklist and questionnaire (19 questions in Dutch). Control group (*n* = 188) and experimental group (*n *= 198)	INVOLVEMENT	The number of process deviations associated with elective IR procedures can be significantly reduced when patients are consulted in an IR outpatient clinic before the procedure. Increase in informed consent for those in the treatment group	*Hospital systems/staff recognizing the role of IRs relating to patient before the intervention. *Recognition of the value of the relationship rather than conducting the procedure only	*Dutch‐speaking participants only. *Barrier relates to research data collection rather than actual intervention
					Appointment 2–14 days before the scheduled IR procedure, information, screening for risk and consent obtained			
Opsahl et al.^41^	2017	USA/hospital	Explore the impact of adding a video educational engagement strategy intervention for patients and families added to the current fall prevention intervention	Staff interview, falls data. A pre/posttest comparison of monthly and quarterly fall rate reports before, during and after video implementation guided the study (*n* = 2148)	CONSULTATION	Video offers the clinician another component to collaborate with patients and their families, and impact patient education outcomes. Including video engagement for the patient can result in positive trends towards a decrease in the fall rate of hospitalized patients	*Staff education, *engaging patients and families with video, *incorporate evidence‐based preventative strategies in all levels of the healthcare system	*Technology and resources
					The addition of an educational video for patients in an existing falls prevention strategy to attain better fall rates			
Rochon et al.^42^	2019	USA/hospital	To describe the process of implementing and developing a falls prevention programme aimed at decreasing falls and improving patient safety by including patients in their care	Used the plan, do, study, act model implementation period from 10 June 2014 to 31 May 2015	CONSULTATION	The rate of falls decreased (71%) from 8.06 to 3.18. The average number of falls decreased from 4 to 1.7. The length of stay also decreased, meaning that the cost to the hospital decreased	*Staff commitment, *dedicated falls prevention role, *video monitoring to reduce falls, *cost reduction due to decreased falls	*Communication with patients about their involvement in the project— patients were unaware that they were involved in the falls prevention project
					‘Partnering with the Patient’ had 4 parts: (1) Engaging the patient. (2) Communicating fall safety goals. (3) Enquiring about safety concerns. (4) Rewarding patients for not falling during their stay			
Seale et al.^36^	2015	AUS/hospital	The purpose of the pilot study was to test hospital patients' acceptance of a new enabling tool designed to increase patient awareness and participation in the prevention of healthcare‐associated infections (HCAIs)	Nineteen questions survey conducted two times: At baseline and after discharge (*n* = 60)	PARTNERSHIP	Participants more likely to ask a factual question than challenge staff about hand hygiene—more likely to challenge staff when more informed; three patients asked staff about hand washing after the intervention	*Being informed about infection control, *importance of verbally delivering the messages rather than just providing written material	*Baseline survey measured intention—this does not always = behaviour, *need empowerment messages only in languages other than English
					Empowerment tools, a Flip chart and a brochure with information about HCAI and the role that the patient plays in preventing them			
Silkworth et al.^37^	2016	USA/hospital/hospital	Describe a staff‐driven quality improvement initiative to develop a video in partnership with patients and families to prevent falls when hospitalized. Engaging patients and their families in a ‘2‐way conversation’ about how they can participate in meeting a mutual goal rather than by one‐sided education	Data collected via EMR about patient response	CONSULTATION	Falls rates have decreased by 29.4% since the release of the video—other measures were implemented simultaneously—for example, hourly rounds and a broader focus on falls prevention‐ attributable to patient engagement	*Learning style	Not identified
					Mandatory video about falls to increase interaction between patients and staff about falls			
van Gaal et al.^39^	2011	The Netherlands/hospital and aged care	To test SAFE or SORRY programme impact on AEs related to ulcers, UTIs and falls	Patient file review, AE data and weekly inspection of patients' skin. A total of 1081 in the intervention group (*n *= 1081) and 1120 usual care in hospital (*n *= 1120). A total of 196 intervention and usual care (nursing home (*n* = 3045)	CONSULTATION	Implementation of multiple guidelines simultaneously is possible. Patients in the intervention groups developed 43% and 33% fewer adverse events compared to the usual care groups in hospitals and nursing homes, respectively	*Tailored education patient involvement and feedback dealing with multiple potential AEs at the same time, *using an approach with many different elements	*Underreporting
					Consisted of education, feedback through a computerized registration programme and an implementation plan for every ward			
Watt et al.^54^	2020	Canada/primary healthcare	Help prevent medication‐related failures and manage opioid use	Feedback was gathered from services (*n* = 200), and opioid study patient survey (*n* = 127)	PARTNERSHIP	The tool enables patients and families to start a conversation about medication. In conjunction with other strategies, researchers noted increased patient education and decreased use of opioids	*Engaged health services. *Further evaluation, *empowered patients, *collaboration	*Limited service/practitioner engagement
					Patients were empowered to ask 5 specific questions about medication for use, staff training and standardized electronic prescribing			

*Note:* Baker et al.^52^ reported on three strategies; two fulfilled the inclusion criteria for this review.

*Consultation*: In the context of Carman et al.'s^10^ framework, nine strategies summarized in Table 1 conceptualize engagement as *consultation*. What is distinct in this phase of engagement is that patients were consulted, or invited to provide input, about a specific safety issue/s within parameters of engagement set by health practitioners.

Four strategies involved staff‐initiated engagement about a specific treatment or potential adverse event. Kim et al.^44^ describe how direct questioning by staff to patients about the site of their X‐ray at an orthopaedic clinic in Korea led to a significant decrease in X‐ray site errors. Bergal et al.^40^ describe a similar strategy implemented to reduce wrong‐site surgery in the United States of America with less definitive findings, primarily due to few incidents of wrong‐site surgery. van Gaal et al.^39^ described a programme focused on reducing poor outcomes by staff providing education and opportunity for engagement in three areas (ulcers, urinary tract infections and falls) in 10 wards across four hospitals and aged care facilities in the Netherlands.^39^ Rochon and Salazar^42^ described a four‐stage falls reduction process implemented in medical/surgical wards in a USA hospital. Although both van Gaal et al.^39^ and Rochon and Salazar^42^ reported decreased falls and fewer adverse events, limited details about interactions between staff and patients were reported. These two interventions have been classified as *consultation* due to the focus on patient education and staff‐directed interaction.^39^, ^42^ One strategy sought to create engagement about a safety event by driving patient‐initiated contributions through a feedback mechanism: the Patient Reporting and Action for a Safe Environment (PRASE) Tool. PRASE was trialled in 33 wards across 5 UK hospitals, which demonstrated a decrease in preventable harm at the ward level.^50^

Four strategies described staff adapting existing engagement tools to promote interaction by staff with patients in hospital settings and were relevant to this category due to the focus on patient education and staff‐directed interaction.^31^, ^37^, ^41^, ^55^ Silkworth et al.^37^ developed a 5‐min video to encourage patients and their families to engage in a ‘2‐way conversation' about falls risks on admission,^37^ and Opsahl et al.^41^ added a video to an existing falls prevention strategy. Both studies were conducted in acute care hospitals in the United States of America and both reported decreased falls and positive findings about using video to engage patients. Similarly, Duckworth et al.^31^ and Dykes et al.^55^ evaluated the addition of a multimodal approach (laminated, electronic or bedside display) to present information of a person‐centred falls prevention plan (FallTIPS) in three large hospitals in the United States of America^31^, ^54^ (see Table 1 for effectiveness data).

*Involvement*: The *involvement* phase of engagement^10^ indicates that patients were asked about their preferences and concerns, with the opportunity to interact and engage with practitioners about a specific health or treatment issue. This stage of engagement contains strategies devised by staff, offering opportunities for increased ongoing interaction between staff and patients that were not evident in strategies classified as *consultation*. Six strategies sought to enhance safety by *involving* patients (Table 1).

Only one strategy in the *involvement* phase of the continuum was related to face‐to‐face interactions between staff and patients, reporting on a strategy used in an outpatient interventional radiology clinic in the Netherlands.^45^ Clinic patients were invited to attend an additional appointment before their interventional radiology visit to discuss their queries and concerns about the procedure, risk and consent, hence the classification as *involvement*. This strategy enhanced the relationship between the practitioner and the patient, led to increased informed consent and a reduction in deviations from process (Table 1). Two further online strategies were used to facilitate communication between patients and health practitioners^35^, ^38^ about specific areas of care. de Jong et al.^38^ evaluated an online medication reconciliation and Heyworth reported on a similar pilot study of recently discharged patients from a USA veterans' hospital and reported that patients notified staff of medication discrepancies with potential for significant adverse reactions.

Three online feedback strategies provided an opportunity for patients to raise issues and interact with staff about safety concerns across their care experience. All three studies were hospital based and conducted in the United States of America.^32^, ^33^, ^52^ Bell et al.^53^ reported on the efficacy of open notes with a feedback tool,^53^ Gerard et al.^33^ explored patient experiences using electronic notes viewable by patients in a hospital setting^33^ and Grossman et al.^34^ reported on portals as a mode to engage with patients about safety. All papers reported positive findings in relation to opportunities for patients to raise concerns, although detailed data were not available about the impact of portals on safety outcomes (see Table 1).

*Partnership/leadership*: Strategies that create a partnership between healthcare providers and patients are at the endpoint of the continuum of engagement.^10^ Almost half of the strategies (12) sought to provide patients with the opportunity to raise concerns about their treatment and ‘work’ with practitioners to improve the safety of their care and treatment, often with strategies using person‐centred tools or designed to empower patients to alert practitioners of concerns.^30^, ^32^, ^36^, ^43^, ^46^, ^47^, ^48^, ^49^, ^51^, ^52^, ^54^ Although all strategies in this classification enhance partnership, only six strategies included patients in the inception, design or evaluation of strategies.^46^, ^47^, ^48^, ^49^, ^52^, ^54^

Of the range of strategies included, six described collaboratively developed tools^46^, ^47^, ^48^, ^49^, ^52^, ^54^ and processes designed to encourage and facilitate patient communication and feedback. All six studies reported positive impacts on patient safety, including decreased adverse events and increased identification of errors that would have resulted in harm (see Table 1). Four of the six strategies were ‘bedside’ tools collaboratively developed with patients designed to enhance quality and included ‘safety’ as one of many goals.^46^, ^47^, ^48^, ^52^ Dykes et al.^46^ evaluated a suite of strategies implemented in two medical intensive care units in the United States of America.^46^ Khan et al.^48^ reported on a patient‐centred project implemented in medical paediatric wards at one Canadian and six US teaching hospitals. Transforming care at the bedside (TCAB) is a codesigned bedside checklist designed to enable families to provide real‐time feedback on various quality measures, including safety, implemented the TCAB in 19 units at 6 hospitals in Montreal, Canada.^52^ Family‐centred rounds evaluated in four US paediatric hospital sites used a similar approach to the strategies described above, concluding that patient‐centred engagement is effective for identifying patient safety concerns.^47^ A bedside safe‐outcomes reporting tool was collaboratively developed to enable patients to measure risk and raise unsafe care issues in an inpatient paediatric renal ward in a United Kingdom.^49^ The tool was pictorial for ease of use and patients recorded concerns as issues arose; this led to an increase in critical incident reporting by staff. The Fracture Recovery for Seniors at Home (FReSH START Toolkit) was collaboratively developed by staff, patients and their families and highlighted the value of collaboration with patients and caregivers in preventing complications.^52^

Three partnership or leadership strategies sought to empower patients to take responsibility for specific elements in their care as inpatients, although they did not report patient involvement in the design or inception of the strategy.^36^, ^51^, ^54^ Seale et al.^36^ report on the use of a flip chart and brochure with the aim of empowering healthcare consumers to take responsibility for safety, and alert staff to hygiene issues in an Australian hospital. Watt et al.^54^ report on a Canadian strategy implemented in 200 community and inpatient healthcare settings that encouraged patients to ask five questions. The aim was to decrease medication errors and the paper reported on a study that showed the impact of this intervention on opioid use. Campbell et al.^51^ reported on a strategy implemented in paediatric intensive care units in Hanoi, Vietnam. The strategy described was a bedside tool to encourage patients to remind staff to wash their hands (see Table 1 for effectiveness data).

The final three strategies of the 12 in this group were designed to assist patients to highlight issues at various points of direct care. A Norwegian developed online tool enabled improved communication between breast cancer patients and multiple practitioners, including communications about medications, and treatment.^43^ The strategy progressed other strategies by providing patients with opportunities to clarify issues at a time of their choosing. Two studies highlighted the benefits of patient‐held medication information.^29^, ^31^ Garfield et al.^32^ evaluated medication reconciliation tools used by people who access primary and secondary healthcare organisations in greater London, UK, and noted the benefits of using patient‐held medication management tools in various formats. Similar conclusions were reached by Buning et al.^30^ in their proof‐of‐concept study exploring a mobile application for medication reconciliation in a Netherlands hospital. These three studies reinforced the need for flexibility and benefits of patient‐managed tools that span various healthcare providers and agencies to increase safety when care spans various sites.

#### 3.1.2 What types of patients and contexts are described in the interventions?

The included studies were predominantly conducted in inpatient settings (13 studies) or after discharge from inpatient stay (10); the remainder were conducted in outpatient clinics or community settings, including an aged care facility (4). Twenty‐four studies were conducted in countries classified as ‘developed’ by the United Nations, 25 studies in countries classified as high income and one developing economy.^51^ Participants were most often recruited from university or teaching hospitals (22), predominantly city based (17), and in the United States of America (13). Twenty‐five studies recruited male and female participants; one study recruited only women.^43^ Participant characteristics and demographic information were reported with varying levels of detail; all studies recruited participants over 16. Information about culture and ethnicity was variable, and only eight papers provided data about culture and language preferences.^31^, ^32^, ^36^, ^46^, ^47^, ^48^, ^50^, ^53^ No papers reported on any other diverse communication needs.

In 24 papers, engagement approaches were available to all eligible patients. Three papers excluded participants not from dominant language groups for methodological reasons.^36^, ^45^, ^50^ In the remaining papers, participation was open to all; however, people who opted to participate were often identified as well‐educated,^33^ insured and^35^, ^46^ computer literate^35^, ^46^ and tended to be from the dominant language group.

Safety engagement strategies described were only available in the dominant language in 19 papers.^16^, ^30^, ^32^, ^33^, ^35^, ^38^, ^39^, ^41^, ^42^, ^43^, ^44^, ^45^, ^46^, ^51^, ^52^, ^53^, ^54^, ^55^ Two papers reported constrained resource‐limited adaptation of information to meet diverse needs of patients, identifying the absence of key groups as a study limitation.^36^, ^50^ Six papers contained commentary about the suitability of tools for CALD communities, noting that more older non‐Caucasian patients accessed the Open‐notes tool than anticipated,^53^ the benefits of a visual tool^49^, ^51^ and highlighting the need for alternative or adapted strategies.^36^, ^40^, ^47^, ^48^, ^49^

The question of the effectiveness of strategies ‘for whom’ is central to realist synthesis. The included studies are robust; however, they also have insufficient data to determine the extent to which vulnerable or minority groups were represented. The combination of limited socio‐cultural data and a lack of description of how engagement tools were adapted or used means that the effectiveness of strategies for patients from CALD communities or other vulnerable groups is difficult to ascertain.

#### 3.1.3 What are the mechanisms that influence the effectiveness of consumer engagement approaches in enhancing safe care and treatment?

The included studies were examined to articulate common factors identified as influencing the success of strategies to engage healthcare consumers in the delivery of safe care and treatment. Acknowledging the limited data about the inclusion of diverse participant groups (see Q2 and Table 1), four common factors were evident.

*Patient–professional collaboration*: Strategies across the continuum of engagement reported the value of opportunities for staff and patients to establish communication,^47^ form partnerships^37^ and emphasized the value of the ‘relationship’.^45^ These findings are reflective of the evidence that underpins person/patient‐centred approaches.^56^ Some staff participants thought that collaborating with patients about safety could have unintended negative consequences for the practitioner/patient relationship.^41^, ^53^

*Pragmatic and user‐friendly*: Ten strategies emphasize the need for simple feedback systems about safety features that are not time consuming,^49^ use plain language,^48^ not solely reliant on text^49^, ^51^ and can be incorporated into existing documentation systems, interactions or portals.^29^, ^43^ Electronic portals and apps need a user‐friendly interface^38^ and focus on relevant safety concerns.^34^, ^35^ Questionnaires were time consuming and not suited to varied communication needs^49^ or distressed patients.^50^

*Promoting confidence and safety proactively*: The benefit of increasing patient confidence or empowering patients underpinned the implementation success of the interventions across the continuum of engagement.^30^, ^36^, ^37^, ^49^, ^51^, ^54^ The advantages of a proactive approach to enhance safe care were emphasized,^49^, ^51^, ^54^, ^55^ along with the need for cultural awareness and sensitivity.^51^

*Organisational sponsorship*: All papers identify the need for an organisational culture that supports transparency and values health consumer input. Staff training, ongoing commitment of resources including practical adjustment of schedules,^37^, ^39^, ^41^, ^42^, ^48^, ^51^, ^54^ staff consistency,^44^ systematic/whole of agency approach^48^ and management support^31^, ^46^, ^52^ were identified as vital for consumer engagement interventions to be implemented effectively.

## 4 DISCUSSION

Our findings identified 27 strategies that used interactive technologies, dedicated additional appointments and verbal communication prompts to engage patients in ensuring safe care and treatment during direct care. Multimodal strategies were also used in several studies. Most of the strategies were implemented in inpatient settings. The strategies were predominantly evaluated in locations characterized by a significant cultural shift towards patient partnership. The included papers were largely from America (13), Northern Europe (3) and the United Kingdom (3).

The nature of engagement across the strategies spanned the patient engagement classifications of consultation (nine strategies), involvement (seven strategies) and partnership (twelve strategies).^10^ Working in partnership with patients and families is central to devising suitable engagement approaches for specific populations or settings.^10^ It was notable that publications provided varied levels of detail in data about the type and degree of patient engagement in strategy development or implementation. In some instances, researchers identified limited inclusion of diverse patients as an issue to address; however, it was difficult for researchers to ascertain whether it was the patient engagement strategy or research data collection tools that precluded engagement.^32^, ^38^, ^45^ While some papers included details about codesigning strategies with patients,^46^, ^47^, ^48^, ^49^, ^52^, ^54^ this aspect of engagement is most often absent, undefined or unreported. Insufficient information about such elements as the patient role in strategy design reflected limited evidence that the strategies described were theoretically informed. Lawton et al.^50^ provided a theoretical background to engagement; similarly, the patient‐centred strategies embedded engagement in such approaches.^46^, ^47^, ^48^ However, the theoretical justification for strategy design presented in most papers was on content (e.g, falls prevention or wrong‐site intervention), technical production (e.g., videos^37^) or staff implementation,^41^, ^43^ rather than on the nature or details of engagement.

Attributing changes in patient safety outcomes to a particular type of patient engagement was challenging due to the variation in the definition of engagement in the included papers, which is reflected in the wider literature.^10^ Lack of consistency in defining ‘engagement’, coupled with limited details about strategy implementation and participant characteristics, created challenges in understanding the types of engagement strategies that work to achieve particular outcomes in particular populations or contexts of care. These shortcomings have been recognized in the literature, with Lawton et al.^50^ concluding that the widely used practice of using adverse events reporting data to ascertain the impact of specific engagement strategies is unsuitable. Similarly, Wright et al.^5^ highlight the challenges to measuring the impact of patient engagement strategies on safety and call for more detailed analysis of engagement.

Knowledge of intervention effectiveness, acceptability and feasibility is critical in the context of emerging evidence of both increased risk of safety events and barriers to engagement for particular patient groups. The needs of patients from ethnic minority backgrounds^23^, ^57^ and disability^24^, ^58^ have been highlighted in recent reviews.^21^ There is emerging literature about the advantage of animation‐ or picture‐based communication for various patient groups including people from ethnic minority backgrounds or with disabilities;^59^, ^60^ yet, only two included papers incorporated visuals to accompany text.^48^, ^50^ While brief commentary about the effectiveness of specific strategies for people from different ethnic backgrounds and the impact of lower health literacy was made, addressing the needs of diverse participant groups was not a focus of the papers reviewed. Our review therefore identified a need for strategies developed and evaluated with consideration of and input from diverse patient population groups, along with evidence of their effectiveness for people from different ethnic backgrounds, age groups, disability status and other critical patient characteristics.

Patient–professional collaboration, user‐friendly strategies, proactive messaging and agency sponsorship were all recognized as enablers of patient engagement. Findings regarding the facilitators of patient engagement between papers in this review were consistent, confirming recent research seeking to empower patients to raise safety issues within a supportive culture.^15^, ^16^, ^22^, ^61^, ^62^ The importance of agency sponsorship of a collaborative culture for engagement has long been emphasized in the change management and person‐centred care literature.^63^ Staff identified that agency support is required to address the potential impact of engagement on the patient/provider relationship and workload.^34^ These concerns are reflective of Park et al.'s^8^ systematic review, which found that staff were aware of the importance of engaging in safety, but were not always confident to do so. A comprehensive approach including a culture of transparency, collaboration and support to implement evidence‐based engagement strategies is required.^62^, ^64^

### 4.1 Implications

Patient engagement interventions are being deployed across health services to promote patient safety despite vast variations in the definitions and conceptualisation of the concept of patient engagement. Few studies have utilized theory‐informed approaches or robust study designs to evaluate current techniques.

There are implications for health services in the challenges posed to scaling and spreading the adoption of potentially useful patient engagement strategies. There is a danger of unintended harmful impacts for those for whom the intervention may not be suitable. There are resource, financial and ethical implications, given the additional time and technologies required by patients and staff to take part in such interventions. This review reinforces the need for a multifaceted approach to patient engagement, incorporating agency culture, practices and appropriate engagement strategies.^9^

Therefore, researchers need to work collaboratively with health services to establish more robust evidence of (a) what the intervention mechanisms are in current strategies and (b) information about (1) the feasibility and acceptability of the strategies for all parties, (2) the end‐users and (3) cost‐effectiveness.

### 4.2 Strengths and limitations

The capacity to explore varied engagement strategies by using a realist synthesis supported by Carman et al.'s^10^ engagement framework provided scaffolding for the review. Use of Carman et al.'s^10^ framework was useful in light of the varied definitions of patient engagement evident in the included papers and the broader literature. Similarly, use of realist synthesis enabled comparison across a disparate group of studies of varying quality and synthesis of information to influence practice.

The findings of this review must be understood in the context of the limitations of the included studies. We identified limited geographical diversity in the countries where the research originated and a lack of studies that sought to compare groups, or samples that were sufficiently powered. By including only published material, valuable insight from nonpublished and nonempirical work may have been missed.^65^ An additional limitation arises from the wide range of terms used to describe patient engagement in safety and the many different types of journals used to house patient safety research. The lack of evidence regarding the theoretical underpinning of the interventional approaches and their intended impact on patient engagement creates barriers to determining the intervention mechanism/s responsible for identified changes. The diverse purposes of papers included also created challenges, particularly papers that reported on a single element of a bigger project, multiple interventions across several sites or safety outcomes reported among a number of interventions carried out simultaneously. The levels of sensitivity and precision of bibliographic databases vary and can also affect the number of articles returned. We used several databases in addition to manual searching to broaden coverage, but there may have been omissions.

## 5 CONCLUSION

Despite the growing number of patient‐centred tools and safety engagement strategies, evidence about use and effectiveness is limited. More details about how they are used and with whom are required to enable patients and practitioners to engage effectively. More clarity is needed to consistently define patient engagement along with further research to determine which strategies are effective. Little evidence exists about people from minority or vulnerable backgrounds in patient safety, which needs to be addressed due to acknowledged disparities in healthcare safety and engagement.

## CONFLICT OF INTERESTS

The authors declare that there are no conflicts of interest.

## AUTHOR CONTRIBUTIONS

Reema Harrison and the CanEngage project team conceived the study. Stephen Mears completed the database search. Two reviewers (Jiadai Li, Benjamin Jones) completed the initial title and abstract review, followed by an independent screening by a third reviewer (Bronwyn Newman). The inclusion criteria were then independently applied to full‐text articles by two reviewers (Bronwyn Newman, Reema Harrison), with disagreements or uncertainty resolved through discussion. Quality was assessed by two reviewers (Bronwyn Newman, Kathryn Joseph). Bronwyn Newman completed writing of the manuscript. Reema Harrison, Holly Seale, Elizabeth Manias, Merrilyn Walton, Ashfaq Chauhan and Kathryn Joseph provided feedback during analysis and reporting and reviewed the manuscript.

## Supporting information

Supporting information.

Supporting information.

## Data Availability

The data that support the findings of this study are available in the Supporting Information Material of this article.
