# Supplementary material for: Do patient engagement interventions work for all patients? A systematic review and realist synthesis of interventions to enhance patient safety
Source: Health Expect. 2021 Aug 25;24(6):1905–23. doi: 10.1111/hex.13343 (PMC8628590; doi:10.1111/hex.13343)
Supplement: Supplementary file 1 — Supporting information. [file HEX-24-1905-s001.docx]

**Supplementary File 1: Interventions encouraging patient engagement in patient safety**

Database(s): **MEDLINE(R) All including Epub Ahead of Print, In-Process & Other Non-Indexed Citations, Daily and Versions(R) 2010- current**
Search Strategy:

| **#** | **Searches** | **Results** |
| --- | --- | --- |
| 1 | (research or program).hw. | 9420895 |
| 2 | Reproducibility of Result/ or "Surveys and Questionnaires"/ | 806237 |
| 3 | (Program Evaluation or validation stud* or Randomized Controlled* or Outcome Assessment* or CrossSectional Studies).ti,ab,hw,kw. | 923990 |
| 4 | (clinical trial or "mixed method" or comparative study or qualitative or controlled clinical trial or evaluation studies or meta analysis or multicenter study or observational study or randomized controlled trial or review* or systematic review or systematic reviews as topic or validation studies).pt. | 5509501 |
| 5 | (systematic review* or benchmark* or intervention stud* or quality improvement* or quality assurance* or intervention strateg* or interventions or strategies or random*).ti,ab,hw,kw. | 2429705 |
| 6 | or/1-5 | 13916385 |
| 7 | Patient Participation/ | 26620 |
| 8 | ((patients adj2 participate) or (patients adj2 participation)).kw,ti,ab,hw. | 3371 |
| 9 | patient* involve*.kw,ti,ab,hw. | 5833 |
| 10 | patient* engage*.kw,ti,ab,hw. | 3489 |
| 11 | patient participant*.kw,ti,ab,hw. | 528 |
| 12 | patient* contribut*.kw,ti,ab,hw. | 1500 |
| 13 | patient* empower*.kw,ti,ab,hw. | 1362 |
| 14 | patient led.kw,ti,ab,hw. | 475 |
| 15 | (patient* adj decision* adj making).kw,ti,ab,hw. | 1148 |
| 16 | (shared decision making adj3 patient*).kw,ti,ab,hw. | 1931 |
| 17 | (self management adj2 patient*).kw,ti,ab,hw. | 2440 |
| 18 | patient willingness.ti,ab,hw,kw. | 253 |
| 19 | patient intentions.ti,ab,hw,kw. | 18 |
| 20 | or/7-19 | 43981 |
| 21 | Adverse Drug Reaction Reporting Systems/ | 7413 |
| 22 | Accident Prevention/ | 9093 |
| 23 | exp Medical Errors/ | 111146 |
| 24 | patient safety.kw,ti,ab,hw. | 41547 |
| 25 | exp Iatrogenic Disease/pc [Prevention & Control] | 24217 |
| 26 | "root cause analysis".kw. or "root cause analysis".sh. | 383 |
| 27 | adverse outcome?.ti,ab,kw,hw. | 27041 |
| 28 | close call?.ti,ab,hw,kw. | 202 |
| 29 | adverse event?.ti,ab,kw,hw. | 152717 |
| 30 | near miss.kw,ti,ab,hw. | 1556 |
| 31 | near misses.kw,ti,ab,hw. | 923 |
| 32 | (iatrogenic or iatrogenesis or misdiagnosis).kw,ti,ab,hw. | 51741 |
| 33 | missed diagnos?s.kw,ti,ab,hw. | 2435 |
| 34 | drug interactions.kw,ti,ab,hw. | 100090 |
| 35 | ((error* or mistake* or fault$) adj2 (medical or medication* or diagnos* or prevent* or detect* or prescri*)).kw,ti,ab,hw. | 78741 |
| 36 | (sentinal event* or hospital mortality or (culture adj safety) or "culture of safety" or never event*).ti,ab,hw,kw. | 56525 |
| 37 | ((adverse or avoidable or preventable or unsafe or undesirable) adj1 (event* or outcome* or complication* or death or effect$ or accident$ or injur$)).ti,ab,hw,kw. | 328323 |
| 38 | or/21-37 | 654551 |
| 39 | 6 and 20 and 38 | 1520 |
| 40 | limit 39 to (abstracts and english language) | 1349 |
| 41 | limit 40 to yr="2010 -Current" | 1018 |
